# Supplementary material for: Extracting the fingerprints of sequences of random rhythmic auditory stimuli from electrophysiological data
Source: PLoS Comput Biol. 2025 Jan 21;21(1):e1012765. doi: 10.1371/journal.pcbi.1012765 (PMC11785292; doi:10.1371/journal.pcbi.1012765)
Supplement: Attachment — Submitted filename: Review_PLOS.pdf [file pcbi.1012765.s001.pdf]

Review of PCOMPBIOL-D-23-01900

"The brain uses renewal points to model random sequences of stimuli"

Understanding how the brain identifies, models and classifies sequences of stimuli is an important open question. In this work, the authors analyse EEG data recorded during a specific stochastic sequence of auditory stimuli (namely, hand claps samba rhythm) via a clustering procedure, showing how the obtained clusters are comparable to those obtained when partitioning with respect to a renewal point in the sequence. Hypothesis tests are then run on different brain regions to test whether this similarity is statistically significant.

This article addresses an important problem, i.e. how the brain categorises random sequences of stimuli. Several aspects require further clarification, motivation or investigation, as detailed below.

1. The introduction is very short, and does not touch important points. Please provide more background, e.g. on the clustering procedures (when/how they were used before), and comment more in details existing relevant results, in particular [5], highlighting the differences between this and that work.

2. The paper often lacks of clarity, justification and "rigorousness/precision". I list a few examples below.

   - How is the model for generating the auditory stimuli chosen? That is, why choosing 1 with probability 0.8 or 0 with probability 0.2, instead of, let us say, 0.7 and 0.3? Please provide a reference for this ([5] may suffice). Also, on Page 3, line 50, I suppose X^{ν}_{n+1} should be set to 0 and not 1?
   - How are the three subsets (Page 3, line 70) chosen? Why does 11 belong to both $\mathcal{E}^{RPF}$ and $\mathcal{E}^{LPF}$? Note that RPF, LPF, OCC are used on Page 3 but only mentioned on Page 6.
   - It would be useful to add a sketch to visualise the data X^{ν}_{n}, the segment $Y^{e,\nu}_{n}$, as the description on Page 3, lines 65-73, is not so clear.
   - Page 4, dissimilarity measure Δ^{δ}. What is π_b? What is $P(b)$? What is b and why $b \in L^2([0,T])$?
   - Page 5, empirical dissimilarity. The authors use $\hat{\Delta}_{m,n}$ as a notation, but neither n nor m are introduced. In particular, n does not appear in the definition of $\hat{F}^{u,B_j}, \hat{F}^{u',B_j}$, while m is only briefly mentioned on line 112.
   - More information are needed to understand lines 110-114. The authors refer to [5], but it would be useful to have this article self-consistent, without requiring a reader to continuously go back to [5] to read it. More information on how to build a dendogram and the hierarchical clustering procedure are needed, as this is crucial for the paper. Also, on line 127, why do the authors select the partition with four clusters?
   - Is the Brownian bridge in Step 1 defined such that $B(-0.05) = B(0.4) = 0$ or what?
   - Some paragraphs are not clear, and it would be beneficial to reformulate them, such as that on Page 3, lines 75-79.

- Please provide more details on the performed statistical inference. What is the null hypothesis? Why the random symmetric matrices are in $\mathbb{R}^{12\times 12}$? Also, would not make sense to generate the random matrices making use of some of the known information, e.g. 2 is periodic with period 4? Please provide a formula for the adjusted Rand index instead of only a reference. How are the p-values on Page 7 computed? Do they correspond to $\hat{p}(\mathcal{E})$? If so, please state that explicitly on Page 5.
- Please comment more on the Sankey diagram and how to interpret.

3. The performed statistical analysis would need to be extended to better support the claims. In particular

   i. What is the impact of the chosen δ on the results? How are the clustering procedure and the corresponding p-values affected by that?

   ii. In the discussion, the authors write "The brain selects a partition of the past of the stimuli sequence with only four clusters, this partition being defined by last occurrence of the strong beat", but the fact of having four clusters was something imposed (Page 5, line 127). How would the results change if the number of clusters is not fixed?

   iii. In the chosen model setting generating the random stimuli, 2 is not only a renewal point but also a "periodic"beat, as it occurs regularly every four auditory units. Can the authors provide an example without isochronous occurrence of the renewal point? This would help discriminating whether the clustering is driven by 2 being a renewal or a "periodic"point.

Specific comments:

- Page 2, line 6. Instead of [1],[2], please write [1,2]. Similarly on Page 4. for [17,18] (and please check throughout the paper).
- Page 2, line 39. The abbreviation EEG has been introduced before, so one may directly use it.
- Page 3, line 46. Please remove "These sequences [...] manner".
- I would probably use $X_t^{[\nu]}$ instead of X_t^{ν} as notation, to avoid the confusion between X_{n+1}^{ν} and X_{n+1}^{i}.
- Page 4, line 93. [12] should be out of the parenthesis.
- Page 6, line 134. Please write "consensus partitions $P^{\mathcal{E}}$.
- Page 6, line 142. Please remove "between the".
- Page 6, line 150. Please write "6, respectively".
- Page 7, caption of Figure 5. Please reformulate it, as the caption is not clear. Moreover, The last term should be $P^{\mathcal{E}_{RPF}}$.
