# Supplementary material for: Extracting the fingerprints of sequences of random rhythmic auditory stimuli from electrophysiological data
Source: PLoS Comput Biol. 2025 Jan 21;21(1):e1012765. doi: 10.1371/journal.pcbi.1012765 (PMC11785292; doi:10.1371/journal.pcbi.1012765)
Supplement: Attachment — Submitted filename: RevisionComments.pdf [file pcbi.1012765.s002.pdf]

Response to the Editors and Reviewers manuscript PCOMPBIOL-D-23-01900

July 5, 2024

1 Response to the Editors

We thank very much the Editors and Reviewers for the thoughtful and constructive suggestions on the manuscript. We carefully addressed all the points raised by the Reviewers and summarized by the Editors. Their insightful criticisms were incorporated into the new version of the manuscript. We hope that this new version complies with the standards of Plos Computational Biology.

In what follows we address all the comments in detail.

The paper is devoted to verify on output EEG data that they are consistent with a certain stochastic model of the input signal, that fits the actual data generation mechanism. This subject of research has undoubtedly a large interest, as testified by the reports of all the referees. Indeed, some of the authors of the paper under consideration have already published a previous paper (number 5 in their reference list), where a more complicate model of the input is assumed, which is still compatible with the generation of the data. Of course various different statistical models can be validated on the same data, but several criteria to choose among them have been proposed. As referee 2 is asking, the authors should compare the results of the two papers. On the basis of this remark, too assertive statements about the conclusions of an experimental work of this kind should be presented with caution, as Referee 1 suggests.

We thank the Editors for raising these issues, which have been addressed in the point-by-point answer to the reviewers. We compared more deeply throughout the manuscript the results obtained in this paper with the previous results obtained [3] and [4]. Also, we were more cautious with the conclusions of our experimental work.

Both referees 2 and 3 are asking for a clearer explanation, with the necessary biological material, of the "partition" of the location of the electrodes in RPF, LPF and OCC (from the mathematical point of view this is not a partition since 11 belongs both to RPF and LPF).

This points were clarified in the methods section of the manuscript's new version.

But a good wealth of doubts by all referees concerns the clustering procedure used in the analysis of the experimental data. First of all the dissimilarity measure suggested by the authors depend on the parameter delta, whose choice seems to fall somewhat from the sky. In addition, the definition of Delta itself seems unclear. What is P? It selects a random b is L^2[0.T] (I suspect here [0,T] is [-0.05, 0.4], correct?), but then this b should be a common parameter of the compared CDF's, but which? Looking at its estimator does not help, since one of the parameter it depends on an n which does not even appear in the definition (see the remarks of referee 2).

We included the missing notation and specifications in the subsection 'Clustering sets of EEG segments'.

Concerning the clustering procedure itself, some further discussion seems needed (referee 2). The question raised by Referee 3 about the aggregation procedure is very natural: isn't it that eta is the square of the number of v's with u and u' in the same cluster? And, more importantly, how the final partition is selected from the consensus dendrogram? It seems that the number of cluster is externally constrained to be 4, which somewhat weakens the conclusion of the analysis. As the last point raised by Referee 1 indicates, it seems to me that we end up with a partition of input strings, not of output chunks.

We included further explanation on the clustering procedure and choice of the number of clusters the subsections 'Clustering sets of EEG segments' and 'Summarizing partitions obtained from each participant'

A serious point raised both by referee 2 and 3 is that the renewal point of the input sequence is happening periodically, which makes impossible to discriminate, on the basis of this experimental protocol, if the results obtained are due to the regeneration or to the periodicity. The Authors should say something on this delicate issue.

We thank the Editors for raising this delicate point. Mayor changes to the manuscript contents were done to comply with this criticism.

2 Response to Reviewer 1

We thank very much Reviewer 1 for the thoughtful and constructive suggestions on the manuscript.

The methodology presented for analyzing the EEG data is novel and holds great potential.

Mayor point Please make a revision on the following point: - From my standpoint, the title seems overly assertive and lacks complete validation based on the presented results. The same when the authors say: "This clustering procedure indicates that the brain uses renewal points in the stochastic sequence of auditory stimuli in order to build a model." "The clusters obtained from the experimental data show that the strong beat of the rhythmic structure is used by the brain to encode the sequence." And similar sentences in the manuscript. I think that it is really very difficult to prove what is stated. I think the results could be interpreted in terms of the strength of the beat rather than being directly linked to higher-order encoding due to the stimulus. I believe more control experiments are necessary to support the authors' assertions, which is why I suggest toning down the interpretation.

We thank Reviewer 1 for raising this important point. Several changes were done throughout the manuscript to comply with this criticism, including changing the manuscript's title and abstract, as well as toning down the interpretation of the results in the discussion session.

Minor points:

Section Clustering sets of EEG segments

- virtual electrode could be replaced with arbitrary electrode.

Done.

- Figures 3, 4 and 6 could be combined into a single figure with three panels.

Done.

- Figure 5 could be included as an auxiliary figure in the Supplementary Material.

We moved Figure 5 to the supporting information.

- The section finishes by saying: "Using a fixed threshold we finally obtain a partition of the sets $\{Yu : uU\}$ of EEG segments, which we denote by C." Please clarify. Do you mean? A threshold is selected in the dendrogram for each condition to create a partition with four clusters. These partitions are denoted by C.

We thank Reviewer 1 for this comment. We clarified in the manuscript that a threshold is selected in the dendrogram for each condition to create a partition with four clusters. These partitions are denoted by $\mathcal{C}$.

3 Response to Reviewer 2

We thank very much Reviewer 2 for the insightful suggestions on the manuscript.

Understanding how the brain identifies, models and classifies sequences of stimuli is an important open question. In this work, the authors analyse EEG data recorded during a specific stochastic sequence of auditory stimuli (namely, hand claps samba rhythm) via a clustering procedure, showing how the obtained clusters are comparable to those obtained when partitioning with respect to a renewal point in the sequence. Hypothesis tests are then run on different brain regions to test whether this similarity is statistically significant.

This article addresses an important problem, i.e. how the brain categorises random sequences of stimuli. Several aspects require further clarification, motivation or investigation, as detailed below.

1. The introduction is very short, and does not touch important points. Please provide more background, e.g. on the clustering procedures (when/how they were used before), and comment more in details existing relevant results, in particular [5], highlighting the differences between this and that work.

We thank Reviewer 2 for this recommendation. The introduction section was modified, both by introducing more background and expanding the contents of the cited references and also, by adding an explanation of the interest and relevance of using a clustering procedure in the context of this work.

2. The paper often lacks of clarity, justification and "rigorousness/precision". I list a few examples below.

• How is the model for generating the auditory stimuli chosen? That is, why choosing 1 with probability 0.8 or 0 with probability 0.2, instead of, let us say, 0.7 and 0.3? Please provide a reference for this ([5] may suffice). Also, on Page 3, line 50, I suppose $X\mu$ n+1 should be set to 0 and not 1?

We apologize for this mistake, which was corrected accordingly.

• How are the three subsets (Page 3, line 70) chosen? Why does 11 belong to both E RPF and E LPF ? Note that RPF, LPF, OCC are used on Page 3 but only mentioned on Page 6.

We thank Reviewer 2 for raising this point. A more in-depth explanation for the electrodes' choice was added to the data acquisition and pre-processing sections. Also, we added further reference in the manuscript to justify the choice of these electrodes..

• It would be useful to add a sketch to visualise the data X_n, the segment Y e, n , as the description on Page 3, lines 65-73, is not so clear.

Figure 1 was introduced to help the reader to visualise these items .

• Page 4, dissimilarity measure . What is b? What is P(b)? What is b and why b L2([0, T])?

We thank Reviewer 2 for indicating this flaw. We clarified the notation in the revised version of the manuscript.

• **Page 5, empirical dissimilarity. The authors use ˆ m,n as a notation, but neither n nor m are introduced. In particular, n does not appear in the definition of Fˆu,Bj , Fˆu 0 ,Bj , while m is only briefly mentioned on line 112.**

We thank the Reviewer for indicating this point. We clarified the notation.

• **More information are needed to understand lines 110-114. The authors refer to [5], but it would be useful to have this article self-consistent, without requiring a reader to continuously go back to [5] to read it. More information on how to build a dendogram and the hierarchical clustering procedure are needed, as this is crucial for the paper. Also, on line 127, why do the authors select the partition with four clusters?**

We thank Reviewer 2 for bringing this up. We briefly explained the clustering procedure and how the dendrogram is constructed. In addition, we incorporated a paragraph on how to choose the number of clusters in the method section.

• **Is the Brownian bridge in Step 1 defined such that $B(-0.05) = B(0.4) = 0$ or what?**

We clarified this point.

Some paragraphs are not clear, and it would be beneficial to reformulate them, such as that on Page 3, lines 75-79.

We have reformulated this paragraph.

• **Please provide more details on the performed statistical inference. What is the null hypothesis? Why the random symmetric matrices are in R 12×12? Also, would not make sense to generate the random matrices making use of some of the known information, e.g. 2 is periodic with period 4?**

We thank Reviewer 2 for bringing this up. We have further explained how we chose the null hypothesis in the manuscript's new version.

Please provide a formula for the adjusted Rand index instead of only a reference.

We incorporated the formula in the manuscripts'new version.

How are the p-values on Page 7 computed? Do they correspond to ˆp(E)? If so, please state that explicitly on Page 5.

The p-values correspond to $\hat{p}(\mathcal{E})$. This was clarified in the manuscript's new version.

• **Please comment more on the Sankey diagram and how to interpret.**

We thank Reviewer 2 for bringing this up. The Sankey diagram is now better explained in the caption of the figure which appears in the Supporting Information.

3. The performed statistical analysis would need to be extended to better support the claims. In particular i. What is the impact of the chosen δ on the results?How are the clustering procedure and the corresponding p-values affected by that? ii. In the discussion, the authors write "The brain selects a partition of the past of the stimuli sequence with only four clusters, this partition being defined by last

occurrence of the strong beat", but the fact of having four clusters was something imposed (Page 5, line 127). How would the results change if the number of clusters is not fixed?

Thank you for raising this point. In the Supporting Information we analised the changes in the p-values for three and five clusters. The results obtained by these analyses reinforce the paper's main conclusions. We also explain in more details how we chose the threshold δ for selecting the number of clusters in the individual partitions.

iii. In the chosen model setting generating the random stimuli, 2 is not only a renewal point but also a "periodic" beat, as it occurs regularly every four auditory units. Can the authors provide an example without isochronous occurrence of the renewal point? This would help discriminating whether the clustering is driven by 2 being a renewal or a "periodic" point.

We thank Reviewer 2 for raising this important point. We agree that the effects of the renewal point and the periodicity cannot be discriminated by the result and changed the discussion to highlight this issue in the new version of the manuscript.

Specific comments: • Page 2, line 6. Instead of [1],[2], please write [1,2]. Similarly on Page 4. for [17,18] (and please check throughout the paper).

Done.

• Page 2, line 39. The abbreviation EEG has been introduced before, so one may directly use it.

Done.

• Page 3, line 46. Please remove "These sequences [...] manner".

Done.

• I would probably use $X_t[\mu]$ instead of X_t^μ as notation, to avoid the confusion between X_{n+1}^μ and X_{n+1}^i.

We changed the notation of sequences of variables to avoid this type of confusion.

• Page 4, line 93. [12] should be out of the parenthesis.

We added the authors name to the citation.

• Page 6, line 134. Please write "consensus partitions P E.

Done.

• Page 6, line 142. Please remove "between the".

Done.

• Page 6, line 150. Please write "6, respectively".

Done.

• Page 7, caption of Figure 5. Please reformulate it, as the caption is not clear. Moreover, The last term should be P ERP F .

Done.

4 Reviewer 3

We thank very much Reviewer 3 for the insightful suggestions on the manuscript.

The article is very interesting but lack many explanations. Let me list them below

- Page 2, Introduction, could you also compare your work to the work of Friston to understand how far it is from your present analysis ?

We thank Reviewer 3 for this comment. The introduction session was modified by introducing more background and expanding the contents of the cited references, including Friston's work.

- page 3, I think there is a problem line 50, I think it is a 0 (see the sequence below)?

We thank Reviewer 3 for pointing out this mistake, we changed it for 0.

- Page 3, on preprocessing, did you also remove ocular artefacts?

We thank Reviewer 3 for raising this question. Participants kept their eyes closed during the experiment. Furthermore, the chosen outlier detection procedure allowed excluding the remaining eye movement artifacts. We added this information in the manuscript's new version.

- Page 3, more importantly, how did you select the set of 8 electrodes ??? what would you find with another set ? Also explain why you focus on RPF LPF and OCC and not other Regions of Interest. By the way, say in full letters what these regions are the first time we see it in the paper.

We thank Reviewer 3 for raising this point. An in-depth explanation for the electrodes' choice was added to the data acquisition and pre-processing sections. Also, we added further reference in the manuscript to justify the choice of these electrodes. Finally, regions of interest were spelled in full letters the first time they were mentioned in the paper.

- Page 4, explain what are $\pi_B(Q)$ and $P(b)$. Say that $\Delta^\delta(Q, Q')$ is not computable in practice. Explain that you are using an estimate of it.

We thank Reviewer 3 for pointing out this. We clarified the notation.

- page 5, the choice of δ and the threshold where you cut your dendrogram seem to be super important for the building of the individual dendrograms. How did you choose these? Not just the values, but why these values? Also do we agree that at this stage, you might have more (or less) than 4 clusters ?

We thank Reviewer 3 for raising this point. In the new version of our paper we explain the choice of δ. Moreover, we included the results of analyzing the number of clusters from an automatic perspective in the supporting information.

- page 5, If I understand qualitatively the grouping, why did you choose $\eta(u, u')$? and not more basically, the number of individuals with u and u' in the same partition of their individual dendrograms?

The notation $\eta(u, u')$ indicates the number of individuals where u and u' belong to the same cluster. This was clarified in the text.

- page 5 again, the end, why forcing 4 clusters ? I understand that you want to compare to P* for you assumption but it might happen that the brain uses less or more, no ? a rule of the thumb might say that we cut the dendogram at the largest jump...

We thank Reviewer 3 for bringing this up. We incorporated a paragraph on how to choose the number of clusters in the method section.

- page 6 and 7, why do you think the RPF partition is different than the LPF partition, is it just some random fluctuations ? is there a biological explanation ?

We address this comparison in the discussion.

- Also there is no comment on the fact that nothing is detected in OCC ? could you explain it from a biological point of view?

The explanation of the lack of a signature of the structure of the sequence of auditory stimuli in the occipital electrodes was added to the discussion.

- I find the last paragraph a bit contradictory with the title. The biological explanation might be that, as you said, the brain follows the regular occurrences, (here the 2)... It just happens that in this case the 2 is also the renewal point of the random sequence, but we could totally imagine a sequence with a cyclic pattern and renewal points that would not coincide. So maybe this experiment is not enough to conclude with respect to renewal point. Or maybe I'm missing some more explanation here ?

We thank Reviewer 3 for raising this important point. Several changes were done throughout the manuscript to comply with this criticism, including changing the manuscript's title and abstract, as giving a reinterpretation of the results in the discussion session.
