# Supplementary material for: Extracting the fingerprints of sequences of random rhythmic auditory stimuli from electrophysiological data
Source: PLoS Comput Biol. 2025 Jan 21;21(1):e1012765. doi: 10.1371/journal.pcbi.1012765 (PMC11785292; doi:10.1371/journal.pcbi.1012765)
Supplement: Attachment — Submitted filename: Review_PCOMPBIOL-D-23-01900_R1.pdf [file pcbi.1012765.s003.pdf]

Review of PCOMPBIOL-D-23-01900-R1

"The brain uses recurrent occurrences to model random sequences of auditory stimuli"

I would like to thank the authors for having carefully addressed my previous comments, in particular with the added literature background and the provided description of the clustering approach. Some of the added material requires further clarification/detail, so I would suggest the authors to address the further open questions listed below.

1. *Introduction.*
   a) The sentence on the Bayesian inversion (Page 1, lines 5-6) is not clear, as any statistical approach consists in "obtaining the parameters of the model from the experimental data", so what is meant by Bayesian inversion here?
   b) The paragraph on the Kullback-Lieber divergence (Page 1, lines 20-27) seems disconnected from the remaining text. The introduction is very short, and does not touch important points. Please provide more background, e.g. on the clustering procedures (when/how they were used before), and comment more in details existing relevant results, in particular [5], highlighting the differences between this and that work.

2. The authors have not yet clarified/explained why the electrode 11 belong to both $\mathcal{E}^{RPF}$ and $\mathcal{E}^{LPF}$? Choosing the same electrode twice in the two subsets may not allow to distinguish their behaviour, as they share some of the data. I think it would be important to have 11 entering only in one of the two subsets, replacing it with a different electrode in the other subset. Also, it may be useful to add a picture/schematic representation of how the electrodes are located, to better visualise their locations with respect to the brain regions. Also, please define the abbreviations RPF, LPF, OCC in the text.

3. *Dissimilarity measure.* All quantities involved are now defined, but there are still some questions.

   i. What is the rationale for choosing P to be a Brownian bridge? What is the intuition/property/foundation beyond this dissimilarity measure? Why not considering other ones to measure the distance between distributions, say e.g. the KL divergence or the Wasserstein distance? Also, please provide a reference for the dissimilarity measure introduced on Page 6 beyond that introduced for its approximation.

   ii. The use of $F_{\pi_b(Q)}$ is not consistent with $\hat{F}_N^{u,B_j}$. Perhaps the authors may use $F^{u,b}$ for the cdf of Q on b, and $\hat{F}_N^{Q,B_j}$ for the corresponding empirical cdf when $b = B_j$?

   iii. Page 7, new added text in red. It'd probably be better to first introduce the clustering procedure and then comment on the choice of the threshold. Also, with respect to the latter, the authors write "[...] the null hypothesis of a Kolmogorov Smirov [...]". a) Could you please state what the null hypothesis is? $Q^u \overset{d}{u} Q^{u'}$? b) Is delta chosen as a critical value yielding a type 1 error of 5%?

   iv. In the definition of $d(C_1, C_2)$, C_1 and C_2 are strings, while Q^u and $Q^{u'}$ are laws. How can that be that $Q^u \in C_1$? Should it be $u \in C_1$?

   v. One line below the definition of $d(C_1, C_2)$, the authors write "[...] the procedure ends when all sets of strings form a cluster". What do you mean by this?

4. *Results.*

   a. Please specify how many strings were classified as in P^* for the LPF and OCC sets, as done for the RPF set.

   b. In the subsection on Page 10, lines 259+, please recall again what the null hypothesis was when reporting the p-values.

   c. Other than reporting the p-values in the case of three and five clusters, could the authors also comment on the type of partitions obtained? That is, can the authors recognise any similar pattern with respect to the strong beat, or anything supporting their claim that the brain uses the symbol 2 to model auditory stimuli sequence?

   d. In the discussion, the authors write that the brain uses only four clusters (Page 11, lines 278-279), with respect to the other partition with 8 elements from [4]. Connected to the point above, I think it would be useful to run some model fitting/validation to see which scenario (3, 4, or 5 clusters) is the most likely given the data. Is there any evidence that the brain uses 4 instead of 3 clusters?

Specific comments:

- I think "in the stochastic sequence of auditory stimuli"on the last line of the abstract is not needed and can be removed.

- Page 4, line 112. $n \in (1, \ldots, N)$ should be $n \in \{1, \ldots, N\}$. One line below, it should be $L^2[-0.05, 0.4]$.

- Page 5, definition of $\mathcal{Y}_N^{\epsilon,u}$. Y is missing, but other than that, it'd make sense to add the definition of n, i.e. $\mathcal{Y}_N^{\epsilon,u} = \{Y_n^\epsilon, n \in 2, \ldots, N : X_{n-2}^n = u\}$.

- Page 5, lines 144-146. What is the connection between this type of cluster/string and the eye artefacts? Also, which distortion?

- Page 5, line 150. The world *Law* is underlined, while I supposed it was meant to be in italic as in the previous version. Also, last line of the page, please write "[...] $Q^{\epsilon,u'}$, shortly denoted by Q and Q', and estimate it [...]"

- Page 7, lines 180-181 can be removed, as they are similar to the text added in the previous subsection. One may start the subsection with "For each ..."

- Caption of Figure 3. "We take the average of these labels...". Is that the average or the sum, as the $\eta(u, u')$ seems the latter?

- Page 8, lines 192-193. I'd remove "Finally, we select the partitions with four clusters"and "and our conjecture P^* suggests four clusters"and add then on line 203 "in our study. As our conjecture P^* has four clusters, we select a partition with four clusters as well, investigating the effect of having three or five clusters in the Supportive Information Section.

- Page 8, line 211 "[...] consensus partitions P^ϵ". One line after, please replace "following null hypothesis"with its definition, e.g. something like "the null hypothesis that the partitions are not identically distributed".

- Page 8, lines 218. The sentence on the null hypothesis is not clear. Please rewrite it.
- In the formula of $ARI(P_1, P_2)$, there is a $P2$ that should be P_2. Also, please use another letter (e.g. c) than b, as b has been used for the Brownian bridge. Also, shouldn't be q instead of m, i.e. $c(q)$ and not $b(m)$, as there are q elements?
- Page 9, lines 222-224. As it is, it is not entirely clear. Do the authors mean something like "finding a partition P_i from the random matrix R_i that is at least as similar to our suggested partition P^* as that P^ϵ obtained from the clustering procedure described in the previous subsection."?
- Page 9, line 227 "[...] of electrodes when imposing four clusters are shown ..."
- Page 9, line 231. as expected $\to$ as proposed in P^*.
- Caption of Figure 4. What do you mean by "The black line ... final clusters"?
