# Supplementary material for: Extracting the fingerprints of sequences of random rhythmic auditory stimuli from electrophysiological data
Source: PLoS Comput Biol. 2025 Jan 21;21(1):e1012765. doi: 10.1371/journal.pcbi.1012765 (PMC11785292; doi:10.1371/journal.pcbi.1012765)
Supplement: Attachment — Submitted filename: report2.pdf [file pcbi.1012765.s004.pdf]

Report of PCOMPBIOL-D-23-01900

Guest associate editor

The fact that Referees 2 and 3 are summarizing their revision by asking again for a Major Revision puts me in a delicate situation. Indeed, despite the referees acknowledge that their previous remarks were properly addressed, mostly of their current remarks concern the same issues raised in the first round. I will try to summarize the main points raised by these two referees, asking at the same time the Authors to make a serious effort to overcome all of them, included those that I am not reporting here for brevity.

1. The general philosophy and the comparison with [5].

The goal of the experiment is finding a trace of the nature of the sequence of auditory stimuli proposed to the subjects in their EEG data. What does it mean? First of all, in the pressent paper w.r.t. [5], a difference trace is sought for, suggesting the use of different statistical tools to analyze the EEG functional data. In the former paper, it is the fact that the sequence is consistent with a contex tree; in the latter is that the sequence periodically propeses a strong beat every 4 instants and in between two subsequent occurrences the distribution of the signal depend only on the distance from the last strong beat. The Authors describe the occurrences of 2 as recurrent (see the comment of Referee 3 on this subject), I would simply say periodic. Hence both models prescribe a partition for the possible past input sequences, with 8 and 4 classes respectively: within each class of the partition the probability of the next input stimulus is constant. The hypothesis which needs to be tested is that also the corresponding output segment has a constant law. In [5] the hypothesis is validated by an empirical tree pruning technique, whereas in the present paper a clustering technique is employed. The context tree model seems to be of much wider applicability, whereas in the model analyzed in the present paper the presence of a periodic event which is also regenerative makes impossible to ascertain which of two characteristics is crucial to determine the brain activity which have been recorded.

2. The clustering algorithm and the individual dendrograms.

There is some confusion in the terminology used by the Authors'. The final goal should be to cluster input triples, not EEG segments, despite each imput triple is associated to a set of EEG segments, recorded at the end of the presentation of the corresponding triple. It may very well be that two identical EEG segments are associated to different input triples which are not estimated to be equivalent. This should be clarified once and for all. There is still a lot of confusion about the dissimilarity measure considered in the algorithm, that the Authors' failed to clarify even in this new version. For any given electrode $e \in E$ and any input triple u let $Q^{e,u}$ be the law of the corresponding EEG segment in $L^2[T_0, T_1]$, and for any $b \in L^2[T_0, T_1]$ they define $\pi_b(Q^{e,u})$ to be the (marginal) law of the scalar product with b and $F_{\pi_b(Q^{e,u})}$ the corresponding Cumulative Distribution Function, which is not random. As suggested by Referee 2, it could be convenient to denoted it by $F^{e,u,b}$. The dissimilarity $\Delta^\delta(Q^{e,u}, Q^{e,u'})$ between $Q^{e,u}$ and $Q^{e,u'}$ is obtained as the probability (in the ensemble of Brownian bridge trajectories b, as suggested by [13]) that the sup norm of the difference between $F^{e,u,b}$ and$F^{e,u',b}$ exceeds the critical threshold δ. By the way, the problem of choosing the threshold concerns Referee 3, who asks to see the results of at least two different choices, giving also a suggestion about the choice. Next this theoretical dissimilarity is estimated by the empirical dissimilarity. This entails not only replacing $Q^{e,u}$ and $Q^{e,u'}$ with empirical distributions over the occurrences of EEG segments in e following the corresponding input triples u and u', but also replacing the Brownian bridge with the uniform distribution over M samples (there is no index N since the number of occurrences of each input triple in the interval $[T_0, T_1]$ is random). All this taken from granted, the Authors' should introduce the appropriate notation as required by Referee 2. It should be remarked that writing averages over the sets $\mathcal{Y}_N^{e,u}$ and $\mathcal{Z}_N^{e,u}$ is misleading, since a certain EEG segment can appear more than once and in this case is should appear several times in a sum, which is not the case with the notation

used by the Authors. Maybe it could be convenient to define the sets of time indices $\{n : X^n_{n-2}\}$ rather than the sets $\hat{\mathcal{Y}}$, where incidentally there is an evident misprint, survived over the two versions. The notation is readily extended to any so-called virtual electrode $\mathcal{E} \subset E$, where the single recording of an electrode e is replaced by an average over the electrodes in $\mathcal{E}$.

3. Consensus partition and the number of clusters.

The aggregation procedure starts from individual partitions, and there is a threshold to be chosen to produce a partition from the dendrograms obtained in 2. I do not see any information about this choice in the paper, as underlined by Referee 3. The definition of the dissimilarity η is also questioned by Referee 3: notice that squaring the dissimilarities should have an impact in the evaluation of empirical p-values. In any case Referee 2 asks for averages rather than counts. In general it seems that the results should be presented in a neater and more complete way, including what happens with a number of clusters different from 4; I don't think there is a need of any kind of Appendix, given the limited amount of informations contained therein. Referee 2 asks for a more understable explanation of the nature of the empirical p-values that are presented, especially the null hypothesis they refer to, which is that the dissimilarities computed among triples are just random numbers with no relation with the auditory stimuli that heve been proposed. As such, small values mean that the partition selected by the algorithm shows an agreement with the model of the stochastic auditory signal, which is not enough not imply that the brain retrieves such a model, since the agreement of $P^{\mathcal{E}}$ with P^*, even if hardly obtainable by pure chance, is not perfect.

4, Specific ponts (in random order).

a. In the definition of asjusted Rand index, m must be q.

b. Concerning the sets $\mathcal{Y}$ and $\mathcal{Z}$, there is no need to shorten the notation putting the comprehension at risk.

c. Remove the sentence with probability 1: there is no need when you refer to a finite set.

d. It is worth making the presentation of the clustering algorithm self-contained, so please clarify the Freiman-Muniz functional depth measure and the Ward linkage criterion, and why they are used.

e. Clarify that chunking is taken from granted, rather than tested, since the analysis concerns only triple of auditory stimuli.

f. The Authors are not testing whether all the possible combinations...but rather which of the possible combinations...had the same law (starting at 302).
