# Supplementary material for: Extracting the fingerprints of sequences of random rhythmic auditory stimuli from electrophysiological data
Source: PLoS Comput Biol. 2025 Jan 21;21(1):e1012765. doi: 10.1371/journal.pcbi.1012765 (PMC11785292; doi:10.1371/journal.pcbi.1012765)
Supplement: Attachment — Submitted filename: ResponseToReviewers.pdf [file pcbi.1012765.s005.pdf]

Review of PCOMPBIOL-D-23-01900-R1: “The brain uses recurrent occurrences to model random sequences of auditory stimuli”

October 21, 2024

Answer to the Guest Editor

We thank the Guest Editor and Reviewers 2 and 3 for their careful and detailed commentary on the manuscript. As requested we did an extensive rewriting of the main text to address the points brought here and by the Reviewers. This included the realization of new analysis procedures which where added to the main text. With respect to the Guest Editor comments:

1. The general philosophy and the comparison with [5].

Thank you very much for raising this point. In this new version of the manuscript, we give a more in detail comparisons between our work and the work in [5]. We also provide a new description of the conclusions which can be inferred from our results. These changes led to a new version of the Title, Introduction and Discussion sections.

2. The clustering algorithm and individual dendrograms.

We thank the Guest Editor for this comment. Following these and the Reviewers' comments, we extended the Methodology and Results sections. These changes included the review of the notation and the inclusion of new definitions on the parameters used in the analysis. We also made an effort to make it clear that the set of strings index a partition of all the EEG segments and therefore no EEG segment will be assigned to more than one dataset.

3. Consensus partitions and the number of clusters.

We thank again the Guest Editor for the comments. As mentioned before we did a careful review of the notation and definitions. This led to an extended section describing the procedure employed to find the consensus partition. In addition, we conducted new analysis to determine the sensitivity of our results to the choice of parameters values and, as requested by a Reviewer, a new section investigating the robustness of our choice of electrodes for the analysis.

4. Specific points (in random order).

We addressed each point individually in the main text. We thank again the Guest Editor for his careful evaluation of our manuscript.

Response to Reviewer 2

I would like to thank the authors for having carefully addressed my previous comments, in particular with the added literature background and the provided description of the clustering approach. Some of the added material requires further clarification/detail, so I would suggest the authors address the further open questions listed below.

1 Introduction

1. The sentence on the Bayesian inversion (Page 1, lines 5-6) is not clear, as any statistical approach consists in "obtaining the parameters of the model from the experimental data," so what is meant by Bayesian inversion here?

   We thank the Reviewer for raising this point. We reformulated this paragraph in the introduction section and added more references to familiarize the readers with the usage of Bayesian approach in neuroscience.

2. The paragraph on the Kullback-Lieber divergence (Page 1, lines 20-27) seems disconnected from the remaining text. The introduction is very short, and does not touch on important points. Please provide more background, e.g., on the clustering procedures (when/how they were used before), and comment more in detail on existing relevant results, in particular [?], highlighting the differences between this and that work.

   We have modified the introduction section in accordance with the Reviewer's comments.

2 Electrode Clarification

The authors have not yet clarified/explained why electrode 11 belongs to both ERPF and ELPF. Choosing the same electrode twice in the two subsets may not allow distinguishing their behaviour, as they share some of the data. I think it would be important to have 11 entering only in one of the two subsets, replacing it with a different electrode in the other subset. Also, it may be useful to add a picture/schematic representation of how the electrodes are located, to better visualize their locations with respect to the brain regions. Additionally, please define the abbreviations RPF, LPF, OCC in the text.

We thank the Reviewer for raising these points. A whole new set of analyses considering 1) a cluster composed by the three central electrodes and 2) two separate clusters with left and right electrode subsets without electrode 11 was performed. Corresponding results are now described in detail in the results session. Furthermore, we added a picture depicting the position of the target electrodes and defined the abbreviations RPF, LPF and OCC, in the Data Acquisition and pre-processing sections.

3 Dissimilarity Measure

All quantities involved are now defined, but there are still some questions.

1. What is the rationale for choosing P to be a Brownian bridge? What is the intuition/property/foundation beyond this dissimilarity measure?We incorporated two lines to explain this idea. The first one is at the end of the first paragraph of EEG data analysis. The other phrase appears after the definition of Δ^δ is given. Why not consider other measures to calculate the distance between distributions, such as the KL divergence or the Wasserstein distance? Thanks for raising this point. We agree that it would be undoubtedly interesting to consider other metrics as Wasserstein of KL divergence. Unfortunately, to the best of our knowledge, there are no goodness-of-fit tests for square-integrable functions. We added a paragraph to the discussion. Please provide a reference for the dissimilarity measure introduced on Page 6 beyond that introduced for its approximation. We cited a reference for the dissimilarity measure.

2. The use of $F_{\pi_b}(Q)$ is not consistent with $\hat{F}^N_{u,B_j}$. Perhaps the authors may use $F_{u,b}$ for the cdf of Q on b, and $\hat{F}^N_{Q,B_j}$ for the corresponding empirical cdf when $b = B_j$? We thank the Reviewer for raising this point. The notation was changed accordingly.

3. Page 7, new added text in red. It'd probably be better to first introduce the clustering procedure and then comment on the choice of the threshold. Also, with respect to the latter, the authors write "[...] the null hypothesis of a Kolmogorov-Smirov [...]". a) Could you please state what the null hypothesis is? b) Is δ chosen as a critical value yielding a type 1 error of 5%? We rearranged this paragraph, explicitly wrote the hypothesis of the goodness of fit test and better explained the role of δ.

4. In the definition of $d(C_1, C_2)$, C_1 and C_2 are strings, while Q_u and Q'_u are laws. How can it be that $Q_u \in C_1$? Should it be $u \in C_1$?

   We thank the Reviewer for raising this point. The notation was clarified in the manuscript.

5. One line below the definition of $d(C_1, C_2)$, the authors write "[...] the procedure ends when all sets of strings form a cluster". What do you mean by this? We rephrased the sentence in a clearer way.

4 Results

1. Please specify how many strings were classified as in P^* for the LPF and OCC sets, as done for the RPF set.

   We added a new analysis to better compare the similarity of the results to the expected partition, and also added more in detail description of the distribution of the strings in the selected partitions.

2. In the subsection on Page 10, lines 259+, please recall again what the null hypothesis was when reporting the p-values.

   We clarified this point by extending the explanation of the methodology.

3. Other than reporting the p-values in the case of three and five clusters, could the authors also comment on the type of partitions obtained? That is, can the authors recognize any similar pattern with respect to the strong beat, or anything supporting their claim that the brain uses the symbol 2 to model auditory stimuli sequence?

   As mentioned before we added a more in-depth description of the distribution of the strings from the clusters obtained in the retrieved partitions.

4. In the discussion, the authors write that the brain uses only four clusters (Page 11, lines 278-279), with respect to the other partition with 8 elements from [?]. Connected to the point above, I think it would be useful to run some model fitting/validation to see which scenario (3, 4, or 5 clusters) is the most likely given the data. Is there any evidence that the brain uses 4 instead of 3 clusters?

   We added the results of the model fitting validation procedures in the main text. We also estimated, for all our analyses, the p-value of obtaining by chance the results as similar as the four cluster partition.

5 Specific Comments

- I think "in the stochastic sequence of auditory stimuli" on the last line of the abstract is not needed and can be removed. Done.
- Page 4, line 112. $n \in (1, \dots, N)$ should be $n \in \{1, \dots, N\}$. One line below, it should be $L^2[-0.05, 0.4]$. Done.
- Page 5, definition of $Y^N_{\epsilon,u}$. Y is missing, but other than that, it'd make sense to add the definition of n, i.e. $Y^N_{\epsilon,u} = \{Y^n_\epsilon, n \in 2, \dots, N : X^n_{n-2} = u\}$. Done.
- Page 5, lines 144-146. What is the connection between this type of cluster/string and the eye artifacts? Also, which distortion? Done.
- Page 5, line 150. The word Law is underlined, while I supposed it was meant to be in italic as in the previous version. Also, last line of the page, please write "[...] $Q'_{\epsilon,u}$, shortly denoted by Q and Q', and estimate it [...]" Done.
- Page 7, lines 180-181 can be removed, as they are similar to the text added in the previous subsection. One may start the subsection with "For each ..." Done.

- Caption of Figure 3. "We take the average of these labels...". Is that the average or the sum, as the $\eta(u, u')$ seems the latter? Done.

- Page 8, lines 192-193. I'd remove "Finally, we select the partitions with four clusters" and "and our conjecture P^* suggests four clusters" and add then on line 203 "in our study. As our conjecture P^* has four clusters, we select a partition with four clusters as well, investigating the effect of having three or five clusters in the Supportive Information Section." We rephrased these lines.

- Page 8, line 211 "[...] consensus partitions P_ϵ". One line after, please replace "following null hypothesis" with its definition, e.g., something like "the null hypothesis that the partitions are not identically distributed". Done

- Page 8, lines 218. The sentence on the null hypothesis is not clear. Please rewrite it. We wrote again this section and explained mode in detail the null hypothesis.

- In the formula of $ARI(P_1, P_2)$, there is a P_2 that should be P_2. Also, please use another letter (e.g., c) than b, as b has been used for the Brownian bridge. Also, shouldn't it be q instead of m, i.e., $c(q)$ and not $b(m)$, as there are q elements? Done, but couldn't find where P_2 should be $P_{2,}$..

- Page 9, lines 222-224. As it is, it is not entirely clear. Do the authors mean something like "finding a partition P_i from the random matrix R_i that is at least as similar to our suggested partition P^* as that P_ϵ obtained from the clustering procedure described in the previous subsection"? We have rewritten this line.

- Page 9, line 227 "[...] of electrodes when imposing four clusters are shown ..." Done.

- Page 9, line 231. as expected $\to$ as proposed in P^*. Done.

- Caption of Figure 4. What do you mean by "The black line ... final clusters"? We better explained this caption.

Response to Reviewer 3

Thank you for taking care of my comments. I'm mostly pleased with the changes, but I still have several problems with the manuscript as it is:

- **Definition of "Recurrent Occurrences":** The main problem is that if I prefer that you do not really refer to renewal points, I have doubts about what you mean by "recurrent occurrences." Could you define it more precisely somewhere, and why is 2 the recurrent occurrence that matches your definition in the sequence (and the only one)?

  We thank Reviewer 3 for highlighting this point, which was clarified in the introduction.

- **Eta Calculation:** I think your $\eta(u, u')$ is not the number of v for which $u \sim u'$, but rather this number squared. Again, why don't you use the number directly?

  We thank the Reviewer for raising this point. We gave it a broader explanation. Now we hope to be clear that the quantity $\eta(u, u')$ is not squared.

- **Procedure Steps and Sensitivity Analysis:** There are at least three steps in your procedure:

  1. You choose a δ.
  2. You choose another threshold for the average over the realizations of the Brownian bridge (line 147). Both of these give you a dendrogram/partition per subject.
  3. You have another procedure to aggregate that over subjects and find another partition for the whole population.

  If I understand correctly, you checked the sensitivity for the third aspect. However, I don't see any discussion on the influence of the first two points. The δ seems to be taken due to the KS distribution, but if you change the 0.05 in lines 142-143 to 0.01 or another value, what does it change? For the other threshold, it doesn't even have a name, yet it seems to be super important for the sequel as well.

  We thank the Reviewer for the pertinent comment. The choice of the traditional threshold 0.05 to determine significance in statistical tests influenced the choice of the value of a series of parameters in our analysis. We now take into account how these choices affect the results. A sensitivity analysis has been incorporated into the main text. In this new version of the manuscript, we hope to have clarified the notation.
