# Supplementary material for: Extracting the fingerprints of sequences of random rhythmic auditory stimuli from electrophysiological data
Source: PLoS Comput Biol. 2025 Jan 21;21(1):e1012765. doi: 10.1371/journal.pcbi.1012765 (PMC11785292; doi:10.1371/journal.pcbi.1012765)
Supplement: Attachment — Submitted filename: Review_PCOMPBIOL-D-23-01900_R2.pdf [file pcbi.1012765.s006.pdf]

Review of PCOMPBIOL-D-23-01900-R1

"Extracting the fingerprints of sequences of random rhythmic auditory stimuli from electrophysiological data."

I would like to thank again the authors for having carefully addressed my comments, and having run additional results on both the consistency of the results for different values of α, β, ν, and the further investigation on a possible lateralization component. I found the article notably improved from its first submission, and I am happy to recommend it for publication, confident that it brings further insights into the brain mechanisms and dynamics.

I have a two minor comments with respect to the new sections/results. I would appreciate if the authors could clarify/update the text accordingly.

1. Page 14, line 347. This is not correct, as the strings, 121, 100 and 000, are clustered together in the LPF and not RPF electrodes of Fig. 5. In fact, all 4 bullet points are also valid for LPF other than the 2nd, as LPF does have a cluster with 012 and 002 but without 001. If one ignores that and only focuses on the cluster with the strong beat, than this is also the same. So overall, is the analysis for CNT close to RPF, as written, or to LPF?

2. On Page 16, you wrote "we note that if we also chose a more conservative significance to 0.1, most results still agree with our conjecture.". As there were no results for $\alpha = 0.1$, I suppose that this is a typo, and "to 0.1" should have been "to 0.01"? If this is the case, I disagree that most of results agree with your conjecture, as one one out of 6 p-values is statistically significant based on Table 2.

List of typos/minor changes

1. Page 2, lines 6-7, I would propose writing "For example, Bayesian inference has been succesfully used to estimate parameters relevant for describing mechanisms in the brain"or something similar.

2. Page 2, line 31. Replace electroencephalographic (EEG) with EEG, as the abbreviation has now been introduced before.

3. Page 2, line 36. By "more economical model", do the authors mean "a computationally less demanding model" or something similar?

4. Page 4, line 119: electodes→ electrodes. One line below, please rewrite as"corresponding to electrodes in the right prefrontal cortical region (RPF), left prefrontal cortical region (LPF) and occipital (OCC) region.

5. On Page 7, the sentence "We call this ... for short)" could be removed.

6. On Page 7, line 186, I'd suggest writing "we consider the Brownian bridge as measure P, as in [7]".

7. Page 9, line 206: in which we replace → replacing. One line below: Finally we finally obtain → Finally we obtain.

8. Page 12, line 292. Please remove "with probability one".

9. Page 13, line 303. The second partition should have index RPF and not LPF.
10. Table 1. Could you perhaps use 3 decimal digits for the p-values, as LPF for 3 clusters as a p-value 0.05 which will not be significative, but you highlighted in blue.
11. Page 15, line 364. Please replace "all below 0.06" with "all below 0.05".
