# Supplementary material for: Extracting the fingerprints of sequences of random rhythmic auditory stimuli from electrophysiological data
Source: PLoS Comput Biol. 2025 Jan 21;21(1):e1012765. doi: 10.1371/journal.pcbi.1012765 (PMC11785292; doi:10.1371/journal.pcbi.1012765)
Supplement: Attachment — Submitted filename: Report.pdf [file pcbi.1012765.s007.pdf]

Report of PCOMPBIOL-D-23-01900

Guest associate editor

It seems that all the referees agree that the paper is in a publishable form. However, they raise three remaining points that the Authors have to take seriously into account, together some with minor points that they should consider as well. From my side there is still a number of points I would like the Authors to take into due account.

Page 2, row 5: The meaning of the sentence is unclear.

Page 4, row 120: Futhermore lacks an r. Please check also the structure of the whole sentence.

Page 7, row 179: To say that $F_{Q,b}$ is the cumulative distribution function of the marginal of Q on b is not acceptable. Please say: the marginal of Q on the scalar product $y \in L^2[T_0, T_1] \mapsto < y, b >$, where b is drawn indpendently from the Gaussian measure P. Concerning the latter, notice that a Brownian bridge depends on a variance parameter that it would be interested to know in the practice how it is fixed.

Page 8, row 198: I do not see any reason for using the shortened notation, which deletes the reference to the strings u and u'.

Page 8, next to the last row: The clustering procedure aims to partition $\mathcal{U}$, not $\{\mathcal{Y}_N^u, u \in \mathcal{U}\}$. The resulting partition of the latter is purely instrumental. Hence the starting partition is made by $\{\{u\}, u \in \mathcal{U}\}$. All the presentation should be changed accordingly, beginning with the definition $d(C_1, C_2) = \max_{u \in C_1, u' \in C_2} \hat{\Delta}^{\delta}_{M,N}(Q^u, Q^{u'})$.

Page 9, row 205: I suggest to use "aggregated" rather than "represented".

Page 9, row 206: In the Kolmogorov-Smirnov threshold cited here $|\mathcal{Z}_N^{u,B}|$ should be doubled, otherwise it does not reduce to the previous formula for the case of identical sample sizes.

Page 9, row 206: Replace "the ν-quantile binomial of a distribution" with "the ν-quantile of a binomial distribution". Moreover in "we select the ... partition" insert the word "coarser", since there are many partitions with this property.

Page 10, row 223: As one of the referees pointed out, the η defined in the display is not what is declared in row 226, which is a more reasonable definition, since it can be easily normalized to get a number between 0 and 1 as required in the description of Figure 4 (by the way, in this description it is better to write average rather than sum average).

Page 11, row 268: I see that the Authors preferred c to b but there is a b left which needs to be corrected into c, in the display above row 267.

Page 12, row 292: I have finally understood what do the Authors mean by saying "silent unit with probability 1". Is it opposite to "constituent silent unit" appearing at row 301? In any case, this should be better clarified: maybe "non random" and "random"?

Page 12, row 301: The two sentences starting at this point are unclear, or at least not precise.

Page 14, row 340: Should we read $\mathcal{E}^{LPF}$ rather than $\mathcal{E}^{RPF}$, as suggested by one of the referees?

Page 15, row 360: The null hypothesiss is that the observed results are obtained by chance, and not the opposite.

Page 15, row 374: I think to say "the brain selects ..." is a bit too much. Maybe "seems to select..." would be preferable.

Page 16, row 409: Do you mean 0.05 rather than 0.1?
