# Supplementary material for: Extracting the fingerprints of sequences of random rhythmic auditory stimuli from electrophysiological data
Source: PLoS Comput Biol. 2025 Jan 21;21(1):e1012765. doi: 10.1371/journal.pcbi.1012765 (PMC11785292; doi:10.1371/journal.pcbi.1012765)
Supplement: Attachment — Submitted filename: 3rdResponse.pdf [file pcbi.1012765.s008.pdf]

Review of PCOMPBIOL-D-23-01900-R1: "Extracting the fingerprints of sequences of random rhythmic auditory stimuli from electrophysiological data."

December 31, 2024

Guest Editor

We thank the Guest Editor and Reviewers 2 and 3 for their careful reading of the manuscript. In case the manuscript is accepted we would like to reveal the Guest Editor name's in the acknowledgments.

We addressed all the points as requested. Concerning the Guest Editor comments:

Page 2, row 5: The meaning of the sentence is unclear.

We changed this line as suggested by the Guest editor.

Page 4, row 120: Futhermore lacks an r. Please check also the structure of the whole sentence.

We rewrote the sentence as also suggested by the Guest Editor.

Page 7, row 179: To say that FQ,b is the cumulative distribution function of the marginal of Q on b is not acceptable. Please say: the marginal of Q on the scalar product $y \in L_2[T0, T1] \to \langle y, b \rangle$, where b is drawn independently from the Gaussian measure P. Concerning the latter, notice that a Brownian bridge depends on a variance parameter that it would be interested to know in the practice how it is fixed.

We changed the notation related to the marginal distribution. Concerning the interest in knowing how the variance parameter is fixed, the new analyses added in the more recent version of this manuscript show consistence concerning the choice of parameters. Anyhow, a comment on the relevance of further exploring this issue in future studies was added in the discussion session. On the final point, we understand that the effect of the choice of Gaussian measure is beyond the scope of this article since the new analysis shows that our results are consistent concerning the choice of parameters. However, we added a comment on the discussion on the relevance of exploring this issue in future studies.

Page 8, row 198: I do not see any reason for using the shortened notation, which deletes the reference to the strings u and u'.

We changed the notation to show u and u' as suggested and mentioned that we are only omitting the $\mathcal{E}$ for simplicity.

Page 8, next to the last row: The clustering procedure aims to partition U, not $\{Y_N^u, u \in U\}$. The resulting partition of the latter is purely instrumental. Hence the starting partition is made by $\{\{u\}, u \in U\}$. All the presentation should be changed accordingly, beginning with the definition $d(C_1, C_2) = \max_{Q \in C_1, Q' \in C_2} \widehat{\Delta}^{\delta}_{M,N}(Q, Q')$..

We changed the text as suggested.

Page 9, row 205: I suggest to use "aggregated" rather than "represented".

Done

Page 9, row 206: In the Kolmogorov-Smirnov threshold cited here —Zu,B N — should be doubled, otherwise it does not reduce to the previous formula for the case of identical sample sizes.

Done.

Page 9, row 206: Replace "the -quantile binomial of a distribution" with "the -quantile of a binomial distribution". Moreover in "we select the . . . partition" insert the word "coarser", since there are many partitions with this property.

Done.

Page 10, row 223: As one of the referees pointed out, the η defined in the display is not what is declared in row 226, which is a more reasonable definition, since it can be easily normalized to get a number between 0 and 1 as required in the description of Figure 4 (by the way, in this description it is better to write average rather than sum average).

As this comment was also brought up by one of the Reviewers we made the changes in the formulation and references to this statistic through the text.

Page 11, row 268: I see that the Authors preferred c to b but there is a b left which needs to be corrected into c, in the display above row 267.

Done.

Page 12, row 292: I have finally understood what do the Authors mean by saying "silent unit with probability 1". Is it opposite to "constituent silent unit" appearing at row 301? In any case, this should be better clarified: maybe "non random" and "random"?

We changed the explanations following the suggestion.

Page 12, row 301: The two sentences starting at this point are unclear, or at least not precise.

As we followed the suggestion of calling the constitutive silent events as non random we believe the text is clearer now.

Page 14, row 340: Should we read $\mathcal{E}^{LPF}$ rather than $\mathcal{E}^{RPF}$, as suggested by one of the referees? Done.

Page 15, row 360: The null hypothesiss is that the observed results are obtained by chance, and not the opposite.

Done.

Page 15, row 374: I think to say "the brain selects ..." is a bit too much. Maybe "seems to select..." would be preferable.

Done.
Page 16, row 409: Do you mean 0.05 rather than 0.1?
We rephrased this sentence.

Reviewer 2

I would like to thank again the authors for having carefully addressed my comments, and having run additional results on both the consistency of the results for different values of α, β, ν and the further investigation on a possible lateralization component. I found the article notably improved from its first submission, and I am happy to recommend it for publication, confident that it brings further insights into the brain mechanisms and dynamics. I have a two minor comments with respect to the new sections/results. I would appreciate if the authors could clarify/update the text accordingly.

1. Page 14, line 347. This is not correct, as the strings, 121, 100 and 000, are clustered together in the LPF and not RPF electrodes of Fig. 5. In fact, all 4 bullet points are also valid for LPF other than the 2nd, as LPF does have a cluster with 012 and 002 but without 001. If one ignores that and only focuses on the cluster with the strong beat, than this is also the same. So overall, is the analysis for CNT close to RPF, as written, or to LPF?

We thank the Reviewer for the comments on our new analysis/results. We changed the bullet points to make clearer that the relevant comparison on our CNT analysis is with the expected P* partition.

2. On Page 16, you wrote "we note that if we also chose a more conservative significance to 0.1, most results still agree with our conjecture.". As there were no results for $\alpha = 0.1$, I suppose that this is a typo, and "to 0.1" should have been "to 0.01"? If this is the case, I disagree that most of results agree with your conjecture, as one one out of 6 p-values is statistically significant based on Table 2.

Thanks for the comment. In our previous text the 0.1 referred and alternative threshold for Table 2, that is, if instead of coloring in blue the values below 0.05 we used 0.1, there would be more significant results. We adapted our text to make this point clearer.

We also considered all the minor points and changed the text accordingly.

Reviewer 3

I'm mostly happy but there is still a problem in the definition of eta. I wrote it below as latex formulas So it is a number to the square and not the number

We thank the Reviewer for rising this issue, which was also raised by the Guest Editor. We changed the notation in hope to better explain our similarity measure following both comments. Our goal is to count the average of participants in which the two elements are clustered together. Our new notation shows that we are not counting each pair of participants twice.
